# Estimating the Impact and Cost of the WHO 2010 Recommendations for Antiretroviral Therapy

**DOI:** 10.1155/2011/738271

**Published:** 2010-11-29

**Authors:** John Stover, Lori Bollinger, Carlos Avila

**Affiliations:** ^1^Futures Institute, 41-A New London Turnpike, Glastonbury, CT 06033, USA; ^2^UNAIDS, Avenue Appia 20, 1211 Genève, Switzerland

## Abstract

In July 2010, WHO published new recommendations on providing antiretroviral therapy to adults and adolescents, including starting ART earlier, usually at a CD4 count of 350 or lower, specific regimens for first- and second-line therapies, and other recommendations. This paper estimates the potential impact and cost of the revised guidelines by first, calculating the number of people that would be in need of antiretroviral therapy (ART) with different eligibility criteria, and second, calculating the costs associated with the potential impact. Results indicate that switching the eligibility criterion from CD4 count <200 to <350 increases the need for ART in low- and middle-income countries (country-level) by 50% (range 34% to 70%). The costs of ART programs only to increase coverage to 80% by 2015 would be 44% more (range 29% to 63%) when switching the eligibility criterion to CD4 count <350. When testing and outreach costs are included, total costs increase by 62%, from US$26.3 billion under the previous eligibility criterion of treating those with CD4 <200 to US$42.5 billion using the revised eligibility criterion of treating those with CD4 <350.

## 1. Introduction

In July 2010, the World Health Organization (WHO) published new recommendations on providing antiretroviral therapy (ART) to adults and adolescents in resource-limited settings that revised the guidelines previously published in 2006. The new recommendations encourage starting ART earlier, usually at a CD4 count of 350 or lower, specifies regimens for first and second line therapies, and contains other recommendations regarding laboratory monitoring and other elements [1]. The revised guidelines were developed based on systematic reviews of the evidence, consultation with key stakeholders, and consideration of the impact and cost of potential changes. This paper describes the model and analysis prepared to examine the potential impact and cost of the revised guidelines.

## 2. Materials and Methods

The analysis consists of two parts: first, we construct a model to calculate the number of people that would be in need of ART with different eligibility criteria, in order to calculate the potential impact of the new guidelines. Second, we calculate the costs associated with the potential impact in order to evaluate the financial implications of the new guidelines. 

**Figure 1:**
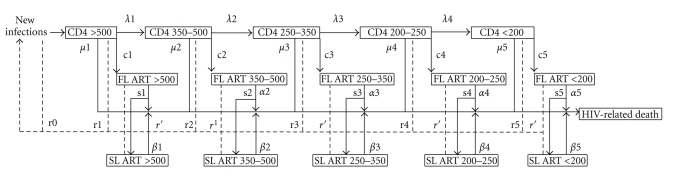
Model of HIV-Infected Population, Eligibility for ART and HIV-related Mortality. Notes: (1) FL ART = First line ART, SL ART = Second line ART, (2) The population receiving ART is categorized according to CD4 count at the initiation of ART, (3) The population in each box is also subject to non-AIDS mortality, and (4) Solid lines indicate flows of people, dashed lines indicate flows of information.

The model tracks the HIV+ population by CD4 count using an approach similar to one used in South Africa recently to estimate the need for treatment (see Figure 1) [2]. The values and sources for all of the parameters described below can be seen in Supplementary Material available online at doi:10.1155/2011/738271 Annex A.

We assume that all newly infected people start with CD4 counts above 500, and that their CD4 counts decline over time. The transition probabilities *λ*1, *λ*2, *λ*3, and *λ*4 represent the probability of progressing from one CD4 category to the next; the derivation of these probabilities is discussed in detail below. In each category there is some probability of death from HIV-related causes, designated as *μ*1, *μ*2, *μ*3, *μ*4, and *μ*5 as well as a chance of death from non-AIDS causes, *μ*0 (not shown in the figure). The probability of HIV-related death increases as CD4 counts decrease.

The number of people in the different CD4 count categories represents the HIV-infected population that is not on ART. The number of people eligible for treatment is the number in each CD4 count category that is below the recommended level for initiating ART. 

Depending on the eligibility criterion and the level of first-line ART coverage a percentage of those eligible for treatment will start first-line ART (c1, c2, c3, c4, c5). Those on ART are categorized by their CD4 count at the initiation of treatment. The model does not track the temporal decline of CD4 counts of those on treatment. Those on first-line ART have a probability of failure depending on their CD4 count at initiation, *α*1, *α*2, *α*3, *α*4, and *α*5.

The number starting ART each year is determined by the assumed coverage and the number of people eligible for treatment. We assume that those starting on ART will be distributed among the eligible CD4 categories such that an equal percentage of people in each eligible CD4 category initiate treatment.

Those failing on first line ART will either start on second line ART (according to second line coverage s1, s2, s3, s4 and s5) or die from HIV-related causes. Those on second line have some probability of dying from HIV-related causes each year (*β*1, *β*2, *β*3, *β*4, *β*5). 

The number of HIV-related deaths each year is the sum of HIV-related deaths from those not on ART and those on ART. 

The historical annual number of new infections is exogenous to the model and is based on a Spectrum projection using historical surveillance and survey data to determine HIV prevalence and incidence trends [3]. The future number of new infections is also based on the Spectrum projection but can be modified by expanding treatment. For those not on ART infectiousness varies by CD4 count (as a result of variations in viral load) as indicated by r1, r2, r3, r4, and r5. Infectiousness is high during primary infection, r0, low during the asymptomatic period (r1, r2, r3, and r4) and high during the symptomatic period, r5. Those on ART have reduced infectiousness, r′. As a result the future number of new infections can be influenced by the dynamics of CD4 decline and the coverage of ART.

## 3. Transition Probabilities

**Figure 2:**
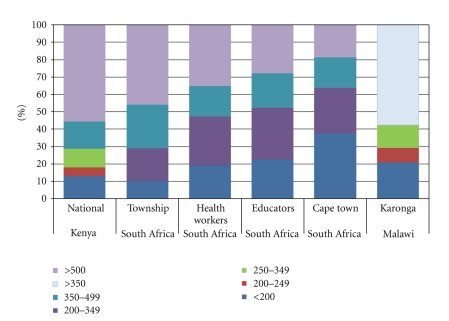
Distribution of HIV+ Population not on ART by CD4 Count.

We have estimated the transition probabilities by fitting the model to data on the distribution of the HIV-infected population by CD4 count and the pattern of progression from HIV infection to AIDS death. Data on the distribution of the HIV-infected populations by CD4 count are available from studies in a township near Johannesburg, South Africa (community-based survey of 1000 men and women aged 15–49 [4]), health care workers in Gauteng, South Africa (all 2032 professional and support staff at two hospitals [5]), educators in South Africa (national survey of 21,669 public school educators from all provinces of South Africa [6]), Cape Town, South Africa (observational cohort from two public sector clinics consisting of 2086 patients [7]), Karonga, Malawi (demographic surveillance site studying all adults aged 18–59 and including about 150 HIV-positive individuals [8]), and Kenya (nationally representative sample of adults 15–64 [9]). The distribution of these populations by CD4 count category is shown in Figure 2. 

Data are also available from several cohort studies on the overall progression from HIV infection to HIV-related death. The Analysing Longitudinal Population-based HIV/AIDS data on Africa (ALPHA) network has conducted a pooled analysis using data from several cohorts to estimate the proportion surviving by the number of years since infection [10]. Only the Kenya data set is a nationally representative sample, and it is the only one that provides information on all CD4 categories of interest. Thus we have estimated the parameter values using only the Kenya data set, along with the age-adjusted, net survival curve based on the East and Southern Africa cohorts from the ALPHA network, but checked the results against the other data sets.

**Figure 3:**
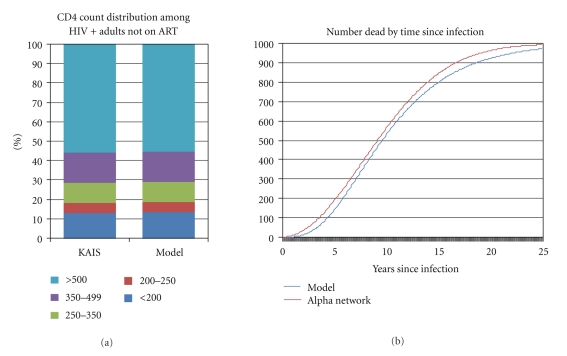
Model results compared to CD4 count distributions in Kenya in 2007 and progression from infection to death compared to ALPHA network analysis.

We fit the model to both sets of data simultaneously. One version of the model was set up for Kenya and used the Spectrum estimates of the number of new infections from 1980 to 2007 and the reported number of people on ART from 2000 to 2007. We compared the data on distribution by CD4 count from the Kenya AIDS Indicator Survey (KAIS) with the model projection for 2007. Another version of the model followed a cohort of 1000 new HIV infections as they progress through the various CD4 categories and to death. The resulting proportions surviving were compared with the ALPHA network survival curve for East and Southern Africa. We searched for the single set of transition probabilities that provided the best fit in both cases. The model used a time step of one-tenth of a year in order to accommodate the short duration in the 200-250 category that could be less than one year. The fits are shown in Figures 3(a) and 3(b). The resulting parameters are shown in Supplementary Annex Table A1. 

**Figure 4:**
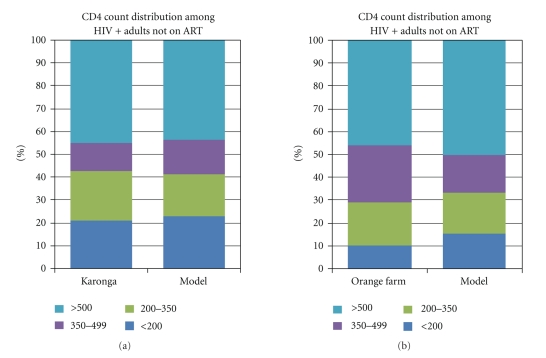
Model results compared to CD4 count distributions for Malawi and South Africa.

The fit of the model to Karonga (Malawi) and Orange Farm (South Africa) data sets using the parameter values derived from the fit to the Kenya data and the annual number of new infections in Malawi and South Africa is shown in Figures 4(a) and 4(b).

## 4. Costs

Four categories of cost are considered: antiretroviral (ARV) drugs, laboratory costs, service delivery costs, and identification (outreach and testing). The cost of ARV drugs is determined from the number of people on first and second line, the distribution of patients by regimen and the costs of each regimen. Following previous work, we examine two sets of alternative regimens: one that contains a fast phase-out of d4T, and another that contains a slower phase-out of d4T [11].

Drug costs may be different for patients in low and middle income countries. Current costs are based on WHO and Clinton Foundation reports (Table 1). 

Laboratory costs are calculated separately for new and continuing patients and can vary by regimen. Currently, laboratory costs are calculated as the annual median cost for lab tests across recent literature. Recent studies in various countries (Cote d'Ivoire, Ethiopia, Mexico, Nigeria, South Africa, Thailand, Uganda, Zambia) are used as the basis [12–20]. The median cost is $250 per year for new patients and $190 per patient per year for continuing patients. 

Service delivery costs are based on a standard number of inpatient days and outpatient visits per patient per year and country specific costs for inpatient days and outpatient visits. For this analysis we used the same studies referenced above for laboratory costs (with the exception of Cote d'Ivoire and the addition of another South Africa study [21]) to calculate the median number of outpatient visits per year as 9.5. Only three of these studies also had data on the number of inpatient days for ART patients [12, 14, 21]; we used these to calculate the median number of inpatient days for ART patients per year as 1.56. The country-specific costs per inpatient day are the costs of one bed day at a primary-level hospital as reported in the WHO-CHOICE database of service delivery costs [22]. The cost of an outpatient visit is for a 20-minute outpatient visit at a health centre, from the same WHO database. Representative regional costs are shown in Table 2.

Outreach and testing costs vary primarily by the type of population reached. The model considers 10 population categories for testing:

1. patients with symptoms of HIV,
2. sexually-transmitted Infection (STI) patients,
3. tuberculosis patients,
4. pregnant women,
5. other health system contacts,
6. sex workers,
7. men who have sex with men (MSM),
8. injecting drug users (IDU),
9. voluntary counseling and testing (VCT),
10. general population.

Due to lack of data, those coinfected with hepatitis B are not included here.

The unit cost of VCT services average about $16 per client. We have used this cost also for provider-initiated testing and counseling. No additional testing and counseling costs are included for pregnant women since the costs of testing and counseling are already covered in the Prevention of Mother-To-Child Transmission (PMTCT) programs. Similarly we assume that outreach and counseling for sex workers, IDU, and MSM are already covered in prevention programs for those populations, and add only $1 for the costs of the test itself. For general population testing we have doubled the personnel costs associated with VCT to allow for additional outreach programs in addition to the testing and counseling costs. The resulting cost is $23 per person tested.

The number of tests for each population group will depend on the eligibility criterion and the coverage. We assume that patients with symptoms who are found to be HIV+ will be in the lowest CD4 count category. We assume that those who are found to be HIV+ in the other population groups will be distributed by CD4 count according to the distribution of all HIV+ people excluding those <200. 

## 5. Results and Discussion

The model has been applied to all low- and middle-income countries (LMIC) and to seven countries individually: Burkina Faso, Mexico, Nigeria, Russia, Tanzania, Ukraine, and Vietnam. The number of new infections each year and the number of people on ART through 2008 were taken from the Spectrum projections for each country. Estimates of the population sizes are based on national estimates prepared as part of the effort to estimate global resource needs [23]. 

Table 3 displays the results for LMIC for the additional cost and impact of scaling up ART coverage to reach 80% by 2015, assuming that the criterion for eligibility to treatment switches from a CD4 count 200 to 350 in 2010. In addition, the financial implications of two ARV regimens are presented; first with a slower phase-out of d4T, and second with a fast phase-out of d4T. In order to compare across countries and across scenarios, all cost and impact figures are discounted to 2010 using an annual discount rate of 3 percent.

Our estimates suggest that switching the eligibility criterion from CD4 count <200 to <350 increases the number of person-years of ART from 40.7 million to over 61 million, a 50% increase. There is a concomitant reduction in the number of AIDS deaths, with the number decreasing by 21% when the eligibility criterion switches to CD4 count <350. The number of new HIV infections is also reduced, due to the lower infectivity that occurs when people receive ART; new HIV infections are reduced by 11% when the eligibility criterion changes.

The financial costs of providing ART to meet the new need from increasing the eligibility criterion also increase in a similar way to the increase displayed in the number of person-years of ART. The costs of providing ART would be 44% higher with a switch to providing ART to those with CD4 count <350. Although the overall costs are higher with the fast phase-out of d4T relative to the slower phase-out of d4T, the difference is quite small. 

Note that the slightly lower percentage increase in costs versus the number of person-years on ART reflects the relatively greater numbers of people on first-line therapy with the increase in eligibility criterion. When the additional testing costs incurred in order to identify the new patients are included, however, total costs increase relatively more than the number of person-years of ART. Total costs increase from US$26.3 billion (US$27.0 billion) to US$42.5 billion (US$43.5 billion) if the eligibility criterion is CD4 count <350 and there is a fast (slower) phase-out of d4T, an increase of 62% (61%). Combining the results for incremental costs and deaths averted suggests that the cost per AIDS death averted is approximately US$9,700 if the eligibility criterion switches to CD4 count <350.

In order to perform a sensitivity analysis, we vary the costs of laboratory testing and service delivery costs using the interquartile distribution of laboratory testing costs from the studies cited above. Using the first quartile function result, laboratory and service delivery costs are reduced by 31%, while using the third quartile function result increases laboratory and service delivery costs by 64%. Overall, this translates to a range in total costs (not presented here) of US$42.5 billion to US$55.5 billion for the scenario with a slower phase-out of d4T, and a range in total costs of US$37.2 billion to US$56.5 billion for a fast phase-out of d4T. 

**Figure 5:**
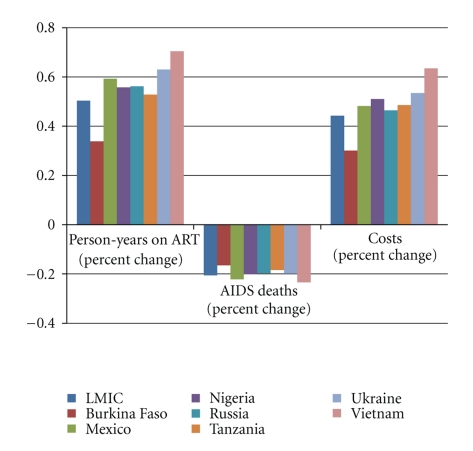
Comparison of results for changing eligibility criterion to CD4 count <350 between LMIC and country-level calculations.

 In order to compare results for different epidemic types and different regions, we performed the analysis for seven countries: Burkina Faso, Mexico, Nigeria, Russia, Tanzania, Ukraine, and Vietnam. Results indicate that there is not a great deal of variation across countries (see Figure 5). While the average percentage increase in the number of person-years on ART for LMIC was 50% when the eligibility criterion switched from CD4 count <200 to <350, this varies across countries from a low increase of 34% in Burkina Faso to a high increase of 70% in Vietnam. A similar pattern can be observed for AIDS deaths; the country level results range from a reduction of 16% in Burkina Faso to a reduction of 23% in Vietnam when the eligibility criterion switches to CD4 count <350. Finally, the changes in the country-level additional ART costs associated with changing the eligibility criterion mirror the changes in the results for LMIC; for LMIC, the additional ART costs increase by 44% when the eligibility criterion switches to CD4 count <350, while the increases at the country level vary from 30% (Burkina Faso) to 63% (Vietnam).

## 6. Conclusions

In this paper, we model both the impact and cost of the new 2010 WHO recommendations for providing antiretroviral therapy to adults and adolescents in resource-limited settings. We examine the impact of changing the eligibility criterion for antiretroviral therapy from CD4 count <200 to CD4 count <350 on the number of person-years on ART, the number of AIDS deaths averted, and the costs of the change including the costs of additional tests and recruitment costs. We also examine the financial impact of switching away from d4T towards other recommended regimens.

We find that, although the total costs for providing ART increase, the percentage increase is slightly less than the increase in number of person-years on ART. The number of person-years on ART increases for LMIC by 50%, varying between 34% and 70% at the country level, while the cost of providing ART increases by 44% for LMIC, varying between 30% and 63% at the country level when the eligibility criterion changes to CD4 count <350. There is minimal impact on the incremental cost when phasing out d4T either fast or more slowly when the eligibility criterion varies. When testing and outreach costs are included, total costs increase by 62%, from US$26.3 billion under the previous eligibility criterion of treating those with CD4 <200 to US$42.5 billion using the revised eligibility criterion of treating those with CD4 <350.

In addition, the number of AIDS deaths decreases at the global level by 21% when the eligibility criterion switches to CD4 count <350, with country-level results varying between decreases of 16% and 23%. Combining the data results in a cost per AIDS deaths averted varying between approximately US$7,100 and US$9,700 (US$4,800 and US$14,000 along with US$6,400 and $16,300) depending on the change in eligibility criterion.

## Figures and Tables

**Table 1:** Antiretroviral costs per patient per year for low- and middle-income countries.

Regimen	Low income countries	Middle income countries
d4T + 3TC + NVP	$89	$88
AZT + 3TC + NVP	$149	$226
AZT + EFV + 3TC	$220	$281
TDF + 3TC + EFV	$210	$268
TDF + FTC + EFV	$255	$325
TDF + FTC + NVP	$190	$243
TDF + 3TC + LPV/r	$590	$1070
AZT + 3TC + LPV/r	$585	$1150

Sources: WHO, UNAIDS and UNICEF, 2009, Towards Universal Access: Scaling up priority HIV/AIDS interventions in the health sector; Clinton Foundation Antiretroviral Price list, August 2009, available at http://www.clintonfoundation.org/files/chaiarvpricelistaugust2009english.pdf, accessed 1 June 2010.

**Table 2:** Representative service delivery costs by region.

Regional service delivery costs for ART patients	Annual cost of inpatient Days (ART patient)	Annual cost of outpatient visits (ART patient)	Total annual service delivery cost (ART patient)
Sub-Saharan Africa	$18.43	$53.62	$72.05
East Asia	$36.48	$64.36	$100.84
Oceania	$56.33	$77.62	$133.94
South and South-East Asia	$29.20	$64.77	$93.98
Eastern Europe and Central Asia	$52.07	$71.82	$123.89
Western and Central Europe	$106.23	$239.38	$345.61
North Africa and Middle East	$63.44	$73.68	$137.12
Caribbean	$58.92	$70.52	$129.45
Latin America	$59.34	$72.91	$132.25

Source: WHO-CHOICE database, available at http://www.who.int/choice/en/.

**Table 3:** Global results when ART eligibility is switched from CD4 count <200 to CD4 count <350 in 2010 while increasing coverage to 80% by 2015, by different d4T phase-out scenarios (2010–2015).

LMIC	CD4 < 200	CD4 < 350	Difference	% Change
Person years of ART	40,752,534	61,292,374	20,539,839	50%
AIDS deaths	8,180,609	6,501,483	−1,679,126	−21%
Life years of PLHIV	162,032,903	163,012,351	979,448	1%
New HIV infections	11,198,013	9,946,912	−1,251,101	−11%
*Slower phase-out of d4T*				
ART costs (Millions of US$)	$25,027	$36,072	$11,045	44%
Testing costs (Millions of US$)	$1,282	$6,480	$5,198	406%
Total costs (Millions of US$)	$26,309	$42,552	$16,243	62%
*Fast phase-out of d4T*				
ART costs (Millions of US$)	$25,678	$37,047	$11,369	44%
Testing costs (Millions of US$)	$1,282	$6,480	$5,198	406%
Total costs (Millions of US$)	$26,960	$43,527	$16,567	61%

Source: Authors' calculations.

## References

[1] WHO Antiretroviral therapy for HIV infection in adults and adolescents: recommendations for a public health approach, 2010 revision. hhttp://www.who.int/hiv/pub/arv/adult2010/en/index.html.

[2] Adam MA, Johnson LF (2009). Estimation of adult antiretroviral treatment coverage in South Africa. *South African Medical Journal*.

[3] Stover J, Johnson P, Zaba B, Zwahlen M, Dabis F, Ekpini RE (2008). The spectrum projection package: improvements in estimating mortality, ART needs, PMTCT impact and uncertainty bounds. *Sexually Transmitted Infections*.

[4] Auvert B, Males S, Puren A, Taljaard D, Caraël M, Williams B (2004). Can highly active antiretroviral therapy reduce the spread of HIV?: a study in a township of South Africa. *Journal of Acquired Immune Deficiency Syndromes*.

[5] Connelly D, Veriava Y, Roberts S (2007). Prevalence of HIV infection and median CD4 counts among health care workers in South Africa. *South African Medical Journal*.

[6] Rehle TM, Shisana O (2005). Estimates of eligibility for antiretroviral treatment (ART) and projected ART impact on AIDS mortality among South African educators. *Journal of Social Aspects of HIV/AIDS*.

[7] Holmes CB, Wood R, Badri M (2006). CD4 decline and incidence of opportunistic infections in Cape Town, South Africa: implications for prophylaxis and treatment. *Journal of Acquired Immune Deficiency Syndromes*.

[8] McGrath N, Kranzer K, Saul J (2007). Estimating the need for antiretroviral treatment and an assessment of a simplified HIV/AIDS case definition in rural Malawi. *AIDS*.

[9] National AIDS (2008). *Kenya AIDS Indicator Survey 2007: Preliminary Report*.

[10] Todd J, Glynn JR, Marston M (2007). Time from HIV seroconversion to death: a collaborative analysis of eight studies in six low and middle-income countries before highly active antiretroviral therapy. *AIDS*.

[11] WHO, Futures Institute, UNAIDS Antiretroviral medicines in low- and middle- income countries: usage in 2008 and a demand forecast for 2010–2012 with a special focus on sub-Saharan Africa.

[12] Bautista SA, Dmytraczenko T, Kombe G, Bertozzi SM (June 2003). Costing of HIV/AIDS treatment in Mexico. *PHRPlus Report*.

[13] Chandler R, Musau R (October 2005). Estimating resource requirements for scaling up antiretroviral therapy in Uganda. *PHRPlus Paper*.

[14] Cleary SM, McIntyre D, Boulle AM (2006). The cost-effectiveness of antiretroviral treatment in Khayelitsha, South Africa—a primary data analysis. *Cost Effectiveness and Resource Allocation*.

[15] Goldie SJ, Yazdanpanah Y, Losina E (2006). Cost-effectiveness of HIV treatment in resource-poor settings—the case of Côte d'Ivoire. *New England Journal of Medicine*.

[16] Huddart J, Furth R, Lyons JV The Zambia Workforce Study: Preparing for Scale-up. http://www.qaproject.org.

[17] Kitajima T, Kobayashi Y, Chaipah W, Sato H, Chadbunchachai W, Thuennadee R (2003). Costs of medical services for patients with HIV/AIDS in Khon Kaen, Thailand. *AIDS*.

[18] Kombe G, Galaty D, Gadhia R, Decker C The Human and Financial Resource Requiremens for Scaling Up HIV/AID Services in Ethiopia.

[19] Kombe G, Smith O (October 2003). The costs of anti-retroviral treatment in Zambia. *PHRPlus Project*.

[20] PHRPlus Rapid Assessment of HIV/AIDS Care in the Public and Private Sectors.

[21] Badri M, Maartens G, Mandalia S (2006). Cost-effectiveness of highly active antiretroviral therapy in South Africa. *PLoS Medicine*.

[22] WHO WHO-CHOICE database. http://www.who.int/choice/costs/en.

[23] UNAIDS Financial Resources Required to Achieve Universal Access to HIV Prevention, Treatment, Care and Support. http://data.unaids.org/pub/Report/2007/jc1678_fin_res_req_en.pdf.

